# Progress of Surgery

**Published:** 1903-02-14

**Authors:** 


*(To be continued.)*

### Progress of Surgery.

### GENITO-URINARY.

**The Surgical Treatment of Chronic Bright's Disease.**—J. A. Schmitt, New York,[1] has contributed a paper on this subject. Tracing the development of the idea of treating chronic Bright's disease by operation, the writer points out how R. Harrison, in 1896, reported a few cases in which he had laid bare the kidneys for supposed calculus or for suppuration, and though no obvious cause for the symptoms—consisting of colicky pains, hæmaturia and albuminuria—was discovered, yet after the operations all the symptoms disappeared. Harrison attributed the symptoms to increased renal tension from congestion and inflammation. J. Israël, in some similar cases, had discovered by operation various inflammatory processes and relieved the patients, but he only recommended operation for intractable symptoms affecting one side. Then G. Edebohls came forward with the statement that chronic Bright's disease was curable by operation, and maintained that renal displacement was one cause of the disease and that fixation of the kidney cured such cases. Later Edebohls advanced the theory that the curative effect of nephropexy was brought about by the resulting adhesive inflammation establishing collateral circulation in the affected organ. A. Pousson had recorded operations performed for various disturbances due to Bright's disease. Schmitt points out that all sorts of kidney diseases in this connection have been mixed up and reported as examples of chronic Bright's disease. He asserts that there is no reasonable ground to suppose that the lesion in true chronic Bright's disease occurs on one side only. A mere surface inspection of a kidney at an operation affords but little information as to the condition of the kidney substance. Kümmell, by ureteral catheterisation had found both kidneys implicated in every instance of chronic Bright's disease he had examined. The author's conclusions may be summed up as follows:—1. Operation, viz., capsular incision, renal cleavage, etc., may be successful in relieving acute kidney disease associated with anuria and uræmic symptoms. 2. Temporary relief has similarly sometimes followed operations in the course of chronic Bright's disease, but permanent cure has never been effected. 3. When the kidney has been operated upon directly for the cure of chronic Bright's disease the outcome has been failure. 4. Renal hæmorrhages and colicky pains in the course of chronic Bright's disease may be successfully relieved by operation. 5. Nephropexy will not cure chronic Bright's disease, though it may relieve albuminuria and other symptoms. G. M. Edebohls (New York)[2] has contributed a paper advocating the performance of renal decapsulation for certain affections of the kidney, rather than nephrotomy, nephrectomy, or resection of the kidney. In decapsulation the kidney is conserved in its entirety and no damage whatsoever is done to its secreting structures. Also the hæmorrhage in the operation is practically nil, and this is often not the case in nephrotomy, nephrectomy or resection. Further, after decapsulation the treatment of the wound is simpler as a rule than in the other operations, for the wound may be closed without drainage, and there is no danger of urinary fistula. In performing renal decapsulation the stripped-off capsule is cut away entirely close to its junction with the pelvis of the kidney. When decapsulation is performed for the purpose of curing chronic Bright's disease, both kidneys should be operated upon at one sitting, for the anæsthetic is more dangerous than the operation. The experiments of Claude and Balthasard, who performed the operation upon dogs, seem to show that the renal function is not appreciably disturbed by renal decapsulation. Edebohls has performed decapsulation on 300 kidneys during nephropexy and on 50 more kidneys for chronic Bright's disease, and states that he has yet to see the first evil effect attributable to the procedure. The author then quotes a series of six cases illustrating the beneficial effects the operation in question is capable of producing. In two of these, one of the kidneys in each case was operated upon and found to be riddled with miliary abscesses, and both patients made good recoveries. One case was of acute hæmorrhagic nephritis and the operation seemed to have cured the disease when the patient died of pneumonia four weeks later. In another case the operation was for intermittent pyonephrosis, but no definite improvement had resulted ten months later.

**Villous Papilloma of the Renal Pelvis.**—L. P. Gamgee [3] records an example of this rare affection, in a man aged 48. His first trouble had been nine and a-half years previously, when he had suddenly felt "something go in his left side," and this had been followed by severe hæmaturia lasting three days. Then for six months there were no symptoms. Then hæmaturia came on again, and the left kidney was exposed with a negative result. For some time after this attacks of hæmaturia recurred at intervals of about six months. A year and a-half later the bladder was opened above the pubes, having become filled with blood-clot, but no source of bleeding could be found in it. After some months the right kidney was explored, also with negative result. The cystoscope had been used, but the source of the bleeding had not been found. Since that time there had been almost constant severe pain in the left loin, and blood had been passed every day. The left kidney was now felt to be much enlarged. Left nephrectomy was therefore performed, and was successful, and the patient was restored to good health. The kidney removed was found to be twice the natural size, with its pelvis dilated and filled with a diffuse villous growth growing from the whole of the pelvic wall. The ureter was not affected. Only a fibrous sac remained in place of the kidney substance. The inner lining of the sac was covered with villous growth over about two-thirds of its area. The miscroscope showed the growth was a non-malignant papilloma. Gamgee remarks that the symptoms of this affection are in the first instance strikingly like those of papilloma in the bladder. Such growths may affect both kidneys. A case has been recorded by Murchison in which both kidneys were affected and the bladder also at the orifice of each urethra.

1 Med. Rec., Sept. 13, 1902, p. 401. 2 Brit. Med. Jour., Nov. 8, 1902, p. 1507. 3 Lancet, Sept. 13, 1902, p. 746.

